# Mathematical foundation of the $U^{N}\left(1\right)$ quantum geometric tensor

**DOI:** 10.1016/j.fmre.2025.04.011

**Published:** 2025-05-02

**Authors:** Xin Wang, Xu-Yang Hou, Jia-Chen Tang, Hao Guo

**Affiliations:** a School of Physics, Southeast University, Nanjing 211189, China; b Hefei National Laboratory, Hefei 230088, China

**Keywords:** Quantum geometric tensor, Mixed states, Geometric phase, Fibre bundle, Quantum distance

## Abstract

In this paper, we systematically establish the mathematical foundation for the ${\text{U}}^{N}\left(1\right)$ quantum geometric tensor (QGT) of mixed states Explicitly, we present a description based on the ${\text{U}}^{N}\left(1\right)$ principal bundle and derive a Pythagorean-like distance decomposition equation. Additionally, we offer a comprehensive comparison of its properties with those of the U(1) principal bundle description of the pure-state QGT. Finally, we prove a fundamental inequality for the ${\text{U}}^{N}\left(1\right)$ QGT and discuss its physical implication.

## 1 Introduction

Over the past few decades, the quantum geometric tensor (QGT) [1], [2] has emerged as a pivotal concept in the study of quantum states, providing valuable insights into their geometric and topological properties. The real part of QGT is the Fubini-Study metric, which quantifies the local distances in the state manifold, while the imaginary part relates to the Berry curvature, linking geometry with topology. Research has shown that the QGT is capable of capturing the sensitivity of quantum systems to fluctuations and local perturbations [3], allowing for the differentiation of various quantum states through its complex structure. Its applications have expanded across multiple fields, including quantum statistical mechanics [4], [5], quantum information [6], [7], condensed matter physics [8], [9], [10], [11], [12], [13], [14], PT-symmetric systems [15], atomic, molecular, and optical (AMO) physics [16], [17], [18], as well as other disciplines [19], [20], [21], [22], [23], [24]. A variety of experimental techniques have been employed to explore the QGT, ranging from Rabi oscillation of an NV center in diamond [25], quench or periodic driving of a superconducting qubit [26], photoluminescence studies of exciton-photon polaritons [27], Bloch state tomography of cold atoms [28], and transmission measurements of plasmonic lattices [29]. Additionally, several other proposals have been put forward to further investigate this novel concept [30], [31], [32], [33].

To date, most studies of the QGT have concentrated on pure states. However, generalizing it to mixed quantum states is both necessary and inevitable, as mixed states are far more prevalent in nature, especially in finite-temperature systems at thermal equilibrium. Recently, there has been growing interest in the geometry and topology of mixed quantum states [34], [35], [36], [37], [38]. In developing the formalism of the QGT for pure states, it is necessary to eliminate extra gauge degrees of freedom, such as the phase factor, because two pure states that differ only by a phase factor are considered physically equivalent. Consequently, the pure-state QGT is invariant under local U(1) transformations. When extending this concept to mixed states, a similar requirement must be met to ensure that the corresponding QGT can properly measure the real distance between inequivalent mixed states. In our previous work (Ref. [39]), we developed a generalized QGT based upon the Uhlmann connection of the mixed-state manifold. Utilizing the purification of density matrices, we extracted the U(N) phase factors of mixed states via the polar decomposition. The mixed-state QGT was then derived by removing the superfluous gauge redundancy from the metric of the purification space. This U(N)-invariant QGT can effectively capture the geometry of the mixed-state manifold via a local distance decomposition [39]. Notably, its real part coincides with the Bures metric, which reduces to the Fubini-Study metric in the zero temperature limit. However, its imaginary part vanishes for ordinary physical processes, a phenomenon that may be attributed to the stringent constraints imposed by U(N) gauge invariance.

In our second approach (Ref. [40]), we proposed an alternative QGT inspired by the Sjöqvist distance [41], [42]. In this framework, the requirement for gauge invariance is relaxed, which may enhance its experimental feasibility. The new QGT is invariant under the local ${\underset{︸}{\text{U}(1)\times \cdots \times \text{U}(1)}}_{N}={\text{U}}^{N}\left(1\right)$ gauge transformation, and we refer to it as the ${\text{U}}^{N}\left(1\right)$ QGT. Its real part is a Riemannian metric, incorporating contributions from both the Fisher-Rao metric and the Fubini-Study metric. Meanwhile, its imaginary part introduces a nonzero U${}^{N}\left(1\right)$-invariant 2-form. Additionally, it exhibits very interesting features in certain physical models [40], [42]. For example, the Sjöqvist distance is closely related to the qubit geodesics, the thermal magnetic susceptibility and the time-energy uncertainty in a unitary evolution. Furthermore, the associated ${\text{U}}^{N}\left(1\right)$ QGT effectively captures the interesting geometric properties of the 2D Dirac-fermion model and the 3D s-wave Fermi superfluid. In this paper, we are going to systematically construct the mathematical foundation of the ${\text{U}}^{N}\left(1\right)$ QGT, presenting a description based on the ${\text{U}}^{N}\left(1\right)$ principal bundle and providing a Pythagorean-like equation for distance decomposition. Moreover, we find that the ${\text{U}}^{N}\left(1\right)$ QGT satisfies a fundamental inequality, similar to that of its pure-state counterpart.

The rest of the paper is organized as follows. In Section 2, we first briefly review the formalism of the pure-state QGT and then develop a systematic description based on the U(1) principal bundle, which facilitates a direct comparison for the later mathematical foundation of the mixed-state QGT. In Section 3, we first present a detailed description of the mathematical foundation of the ${\text{U}}^{N}\left(1\right)$ QGT. Then, we prove a fundamental inequality, and discuss its physical implication. We also illustrate three examples exhibiting different properties of the ${\text{U}}^{N}\left(1\right)$ QGT. In the end, we conclude our findings in Section 4.

## 2 The quantum distance and QGT for pure states

### 2.1 Basic formalism

The concept of physical distance between quantum states is both intriguing and significant in modern physics. Even when considering pure quantum states, this problem is more complex than one might initially expect. We examine a family of normalized states |ψ(R)〉, which depends on a set of continuous real parameters $R={\left(R^{1},R^{2},\ldots ,R^{k}\right)}^{T}$, where R spans the parameter manifold M. A typical example of M is the Brillouin zone of a lattice system. Here, R can be viewed as a local coordinate. Consequently, the (squared) local distance between quantum states with respect to variations in |ψ(R)〉 is

(1) $$\begin{array}{cc} ds^{2} & =∥\psi \left(R+dR\right)\rangle -{\left|\psi \left(R\right)\rangle \right|}^{2}\\ & =\sum_{\mu \nu }\langle \partial_{\mu }\psi |\partial_{\nu }\psi \rangle dR^{\mu }dR^{\nu }. \end{array}$$

However, this is not a proper definition of quantum distance since it is not invariant under the local U(1) transformation: $|\psi \left(R\right)\rangle \to e^{i\chi (R)}|\psi \left(R\right)\rangle$. Hence, we refer to it as the ‘raw distance’ between quantum states. A straightforward evaluation reveals that the distance changes as follows:

(2) $$\begin{array}{cc} ds^{2} & \to ds^{\prime 2}=(\langle \partial_{\mu }\psi |\partial_{\nu }\psi \rangle -i\omega_{\mu }\partial_{\nu }\chi \\ & -i\omega_{\nu }\partial_{\mu }\chi +\partial_{\mu }\chi \partial_{\nu }\chi )dR^{\mu }dR^{\nu }, \end{array}$$

where

(3) $$\begin{array}{c} \omega_{\mu }=\langle \psi |\partial_{\mu }\psi \rangle =-\langle \partial_{\mu }\psi |\psi \rangle . \end{array}$$

Interestingly, $\omega_{\mu }$ is the well-known Berry connection, which transforms like a gauge potential under this U(1) transformation: $\omega_{\mu }\to \omega_{\mu }^{\prime }=\omega_{\mu }+i\partial_{\mu }\chi$. Therefore, we can redefine a ‘proper quantum distance’ based on the properties of the gauge potential [1]:

(4) $$\begin{array}{cc} ds^{2} & =\left(\langle \partial_{\mu }\psi |\partial_{\nu }\psi \rangle +\omega_{\mu }\omega_{\nu }\right)dR^{\mu }dR^{\nu }\\ & =\left(\langle \partial_{\mu }\psi |\partial_{\nu }\psi \rangle -\langle \psi |\partial_{\mu }\psi \rangle \langle \partial_{\nu }\psi |\psi \rangle \right)dR^{\mu }dR^{\nu }. \end{array}$$

In this way, the change of $\omega_{\mu }$ compensates for the change in the original $ds^{2}$ in Eq. (2), ensuring that the new distance is gauge invariant. The corresponding metric

(5) $$\begin{array}{c} Q_{\mu \nu }=\langle \partial_{\mu }\psi |\partial_{\nu }\psi \rangle +\omega_{\mu }\omega_{\nu }=\langle \partial_{\mu }\psi |(1-|\psi \rangle \langle \psi |)|\partial_{\nu }\psi \rangle \end{array}$$

is also referred to as the quantum geometric tensor (QGT) as it precisely captures the local geometric properties of quantum states.

### 2.2 Fibre bundle description

The previous discussion can be systematically framed within the context of a U(1) principal bundle, which provides a clear insight into the geometrical origin of the correction (4). Suppose that all unnormalized quantum states under consideration span an N-dimensional Hilbert space H. The normalized states then form a unit sphere S(H) in H. Topologically, S(H) is equivalent to $S^{2N-1}$, the unit sphere of real dimension 2N−1. Different from the previous discussion, we use the tilde symbol $|\tilde{\psi }\rangle$ to label a normalized state. Two states are considered physically equivalent if they differ only by a U(1) phase factor, i.e. $|{\tilde{\psi }}_{1}\rangle ≃|{\tilde{\psi }}_{2}\rangle$ if $|{\tilde{\psi }}_{1}\rangle =e^{i\chi }|{\tilde{\psi }}_{2}\rangle$. The reason why the distance (1) fails is that it still gives a non-zero result for two physically equivalent quantum states. By factoring out the phase factor, we obtain the equivalence classes of normalized states. Here, we use the untilded symbol |ψ〉 to denote a representative of the equivalence class $\left[|\tilde{\psi }\rangle \right]$, where $|\tilde{\psi }\rangle =e^{i\theta }|\psi \rangle$ for some $e^{i\theta }\in$U(1). If $|\psi_{1}\rangle \neq |\psi_{2}\rangle$, they represent two physically inequivalent states. Consequently, all such states form the quotient space $S^{2N-1}/\text{U}\left(1\right)=CP^{N-1}$, which is the well-known Hopf fibration with $CP^{N-1}$ being the (N−1)-dimensional complex projective space. We also refer to $CP^{N-1}$ as the quantum phase space since all extra degrees of freedom have been eliminated. Note that Eqs. (1) and (4) actually represent the distances on $S^{2N-1}$ and $CP^{N-1}$, respectively. Then, what is the relation between them? Here, we present the fibre bundle description that underlies this relationship.

We take $S^{2N-1}$ as the total space, and $CP^{N-1}$ as the base manifold. A canonical projection connecting them is given by $\pi :S^{2N-1}\to CP^{N-1}$, which collapses all phase factors to project out the physically inequivalent classes of states: $\pi (|\tilde{\psi }\rangle )\equiv \pi (e^{i\theta }|\psi \rangle )=|\psi \rangle$. Conversely,

(6) $$\begin{array}{c} \pi^{-1}(|\psi \rangle )=\{e^{i\theta }|\psi \rangle \left|e^{i\theta }\in \text{U}\left(1\right)\right\} \end{array}$$

represents the fibre space $F_{|\psi \rangle }$ at the point $|\psi \rangle \in CP^{N-1}$. For arbitrary $|\tilde{\psi }\rangle \in S^{2N-1}$, the right action $R_{|\tilde{\psi }\rangle }$ maps g∈U(1) to: $R_{|\tilde{\psi }\rangle }\left(g\right)=|\tilde{\psi }\rangle g=g|\tilde{\psi }\rangle \in \pi^{-1}\left(\pi (|\tilde{\psi }\rangle \right))=F_{|\psi \rangle }$. This implies $R_{|\tilde{\psi }\rangle }\left[\text{U}\left(1\right)\right]=F_{|\psi \rangle }$. Since the right action is a one-to-one mapping, the fiber space is then isomorphic to the structure group U(1), indicating that this fibre bundle is a principal bundle. A smooth map $\sigma :CP^{N-1}\to S^{2N-1}$ given by $\sigma |\psi \left(R\right)\rangle =e^{i\theta (R)}|\psi \left(R\right)\rangle$ is called a section, which satisfies π∘σ=id. Clearly, a section locally fixes the phase factor of a state |ψ〉. Under the current terminology, the connection defined by Eq. (3) is, in fact, a U(1) connection on the total space $S^{2N-1}$, given by $\omega =\langle \tilde{\psi }|d|\tilde{\psi }\rangle$. Substituting $|\tilde{\psi }\rangle =e^{i\theta }|\psi \rangle$, we obtain

(7) $$\begin{array}{c} \omega =id\theta +A=i(d\theta -iA), \end{array}$$

where idθ is a connection on the fibre space, and A=〈ψ|d|ψ〉 is the Berry connection on the base manifold $CP^{N-1}$. To understand the role of ω, we consider a smooth loop R(t) in M, where 0≤t≤τ and R(0)=R(τ). It introduces a loop γ(t):=|ψ(t)〉≡|ψ(R(t))〉 in $CP^{N-1}$. The section σ defines a lift of γ as $\tilde{\gamma }\left(t\right):=|\tilde{\psi }\left(t\right)\rangle =\sigma (|\psi \left(t\right)\rangle )=e^{i\theta (t)}|\psi \left(t\right)\rangle \equiv e^{i\theta (R(t))}|\psi \left(R\left(t\right)\right)\rangle$, which satisfies $\pi \circ \tilde{\gamma }=\gamma$. Note that $\tilde{\gamma }\left(t\right)$ is not necessarily a closed curve, as generically θ(0)≠θ(τ). $|\tilde{\psi }\left(t\right)\rangle$ is said to be parallel transported if it satisfies the condition

(8) $$\begin{array}{c} \langle \tilde{\psi }\left(t\right)|\frac{d}{dt}|\tilde{\psi }\left(t\right)\rangle =0\Rightarrow i\frac{d\theta (t)}{dt}+\langle \psi \left(t\right)|\frac{d}{dt}|\psi \left(t\right)\rangle =0, \end{array}$$

which ensures that $|\tilde{\psi }\left(t+dt\right)\rangle$ remains in phase with $|\tilde{\psi }\left(t\right)\rangle$. Solving this equation, we get the Berry phase at the end of the transportation: $\theta \left(\tau \right)=i\int_{0}^{\tau }dt\langle \psi \left(t\right)|\dot{\psi }\left(t\right)\rangle$, which is invariant under the U(1) structure group. In this context, we refer to $\tilde{\gamma }$ as the horizontal lift of γ. Let $\tilde{X}$ and X be the tangent vectors to $\tilde{\gamma }$ and γ, respectively. The vector X is the pushforward of $\tilde{X}$ by π: $X=\pi_{*}\tilde{X}$. Furthermore, the parallel-transport condition (8) can be reexpressed as

(9) $$\begin{array}{c} \omega (\tilde{X})=0. \end{array}$$

In other words, $\tilde{X}$ is the horizontal vector that belongs to the horizontal subspace of the tangent space $TS^{2N-1}$, and the one-form ω decomposes $TS^{2N-1}$ into its vertical and horizontal subspaces. Therefore, ω is the Ehresmann connection on $S^{2N-1}$. The Berry connection is the pullback of ω by σ: $A=\sigma^{*}\omega$.

Building on this foundation, we can now understand the geometric basis underlying the gauge correction to the distance discussed in the previous section. As indicated by Eq. (1), the distance in the total space can beseparated into

(10) $$\begin{array}{cc} ds^{2}\left(S^{2N-1}\right) & =\langle d\tilde{\psi }|d\tilde{\psi }\rangle =\langle d\psi |d\psi \rangle -2id\theta A+{\left(d\theta \right)}^{2}\\ & =\langle d\psi |d\psi \rangle +A^{2}-{\left(id\theta +A\right)}^{2}\\ & =ds^{2}\left(CP^{N-1}\right)+{\left|\omega \right|}^{2}, \end{array}$$

where

(11) $$\begin{array}{ccc} ds^{2}\left(CP^{N-1}\right) & = & \langle d\psi |d\psi \rangle +A^{2}\\ & = & \sum_{\mu \nu }\left(\langle \partial_{\mu }\psi |\partial_{\nu }\psi \rangle +A_{\mu }A_{\nu }\right)dR^{\mu }dR^{\nu }\\ & = & \sum_{\mu \nu }\langle \partial_{\mu }\psi |(1-|\psi \rangle \langle \psi |)|\partial_{\nu }\psi \rangle dR^{\mu }dR^{\nu } \end{array}$$

denotes the local distance on $CP^{N-1}$, and ${\left|\omega \right|}^{2}=-{\left(id\theta +A\right)}^{2}$ represents the local distance on the fibre space. Note that the absolute-value sign appears because ω, idθ and A are all purely imaginary. Eq. (10) provides a Pythagorean-like decomposition of the local distance in the total space. When a quantum state is parallel transported along a smooth curve, it can be found that $ds^{2}\left(S^{2N-1}\right)=ds^{2}\left(CP^{N-1}\right)$, indicating that the local distance in the fiber space does not contribute to the total distance. Equivalently, the minimization condition of $ds^{2}\left(S^{2N-1}\right)$ is the parallel-transport condition (9). This result is intuitive, as no instantaneous phase is generated during parallel transport. From Eq. (11), it follows that the metric tensor on the base manifold reproduces the gauge-invariant QGT in Eq. (5):

(12) $$\begin{array}{c} Q_{\mu \nu }=\langle \partial_{\mu }\psi |\partial_{\nu }\psi \rangle +A_{\mu }A_{\nu }=\langle \partial_{\mu }\psi |(1-|\psi \rangle \langle \psi |)|\partial_{\nu }\psi \rangle . \end{array}$$

Its real part is the well-known Fubini-Study metric: $g_{\mu \nu }^{\text{FS}}=\frac{1}{2}\left(\langle \partial_{\mu }\psi |\partial_{\nu }\psi \rangle +\langle \partial_{\nu }\psi |\partial_{\mu }\psi \rangle \right)-\langle \partial_{\mu }\psi |\psi \rangle \langle \psi |\partial_{\nu }\psi \rangle$, $\Omega_{\mu \nu }=\frac{i}{2}\left(\langle \partial_{\mu }\psi |\partial_{\nu }\psi \rangle -\langle \partial_{\nu }\psi |\partial_{\mu }\psi \rangle \right)=\frac{i}{2}F_{\mu \nu }$ is proportional to the Berry curvature.

## 3 The Sjöqvist distance and the U${}^{N}\left(1\right)$ quantum geometric tensor

### 3.1 Overview of the basic formalism

The U${}^{N}\left(1\right)$ quantum geometric tensor (QGT), also known as the Sj$\ddot{\text{o}}$qvist QGT, is derived from the Sjöqvist quantum distance between full-ranked density matrices. Similarly, we consider a smooth path $R\left(t\right)={\left(R^{1}\left(t\right),R^{2}\left(t\right),\ldots ,R^{k}\left(t\right)\right)}^{T}$ in M. This introduces an evolving mixed state ρ(t)≡ρ(R(t)). Let |n(t)〉 (n=0,1,… N−1) be the instantaneous eigenstate of ρ(t). Then, ρ(t) can be diagonalized as $\rho \left(t\right)=\sum_{n=0}^{N-1}\lambda_{n}\left(t\right)|n\left(t\right)\rangle \langle n\left(t\right)|$. Following Ref. [42], we further introduce N spectral rays $\left\{e^{i\theta_{n}\left(t\right)}|n\left(t\right)\rangle \right\}$ (n=0,1,… N−1) along the path R(t) and let $B\left(t\right)={\left\{\sqrt{\lambda_{n}\left(t\right)}\{e^{i\theta_{n}\left(t\right)}|n\left(t\right)\rangle \right\}}_{n=0}^{N-1}$ be the spectral decomposition along the path. The Sj$\ddot{\text{o}}$qvist distance is defined as the minimum distance between B(t) and B(t+dt):

(13) $$\begin{array}{ccc} d_{\text{S}}^{2}\left(t+dt,t\right) & = & \underset{\theta_{n}}{\inf}\sum_{n=0}^{N-1}|\sqrt{\lambda_{n}\left(t+dt\right)}e^{i\theta_{n}\left(t+dt\right)}|n\left(t+dt\right)\rangle -\sqrt{\lambda_{n}\left(t\right)}e^{i\theta_{n}\left(t\right)}{\left|n\left(t\right)\rangle \right|}^{2}\\ & = & 2-2\sup\sum_{n}\sqrt{\lambda_{n}\left(t\right)\lambda_{n}\left(t+dt\right)}\left|\langle n\left(t\right)|n\left(t+dt\right)\rangle \right|\{{\dot{\theta }}_{n}\left(t\right)dt\\ & & +\;\arg\left[1+\langle n\left(t\right)|\dot{n}\left(t\right)\rangle dt\right]+O\left(dt^{2}\right)\}. \end{array}$$

The infimum is taken among all possible sets of spectral phases $\left\{\theta_{n}\left(t\right),\theta_{n}\left(t+dt\right)\right\}$, subject to the condition ${\dot{\theta }}_{n}\left(t\right)dt+\arg\langle n\left(t\right)|n\left(t+dt\right)\rangle =0$. Since $\arg\langle n\left(t\right)|n\left(t+dt\right)\rangle \approx -i\langle n\left(t\right)|\dot{n}\left(t\right)\rangle dt$, the minimization condition is equivalent to

(14) $$\begin{array}{c} i{\dot{\theta }}_{n}\left(t\right)+\langle n\left(t\right)|\dot{n}\left(t\right)\rangle =0,\;\text{for}\;n=0,\ldots ,N-1, \end{array}$$

which corresponds precisely to the parallel transport condition for each individual pure state in the ensemble. Under this condition, the Sjöqvist distance becomes

(15) $$\begin{array}{cc} d_{\text{S}}^{2}\left(t,t+dt\right)= & \sum_{n}\left[\frac{{\dot{\lambda_{n}}}^{2}}{4\lambda_{n}}+\lambda_{n}\langle \dot{n}\left|(1-|n\rangle \langle n|)\right|\dot{n}\rangle \right]dt^{2}, \end{array}$$

where the following conditions have been applied: $\sum_{n}{\dot{\lambda }}_{n}=\sum_{n}{\ddot{\lambda }}_{n}=0$, $\langle n|\dot{n}\rangle +\langle \dot{n}|n\rangle =0$, and $\langle n|\ddot{n}\rangle +\langle \ddot{n}|n\rangle =-2\langle \dot{n}|\dot{n}\rangle$. In terms of the parameters R, the Sjöqvist distance can also be expressed as $d_{\text{S}}^{2}\left(R,R+dR\right)=Q_{\mu \nu }^{\text{S}}dR^{\mu }dR^{\nu }$ with

(16) $$\begin{array}{c} Q_{\mu \nu }^{\text{S}}=\sum_{n}\left[\frac{\partial_{\mu }\lambda_{n}\partial_{\nu }\lambda_{n}}{4\lambda_{n}}+\lambda_{n}\langle \partial_{\mu }n|(1-|n\rangle \langle n|)|\partial_{\nu }n\rangle \right]. \end{array}$$

The first term represents the Fisher-Rao metric, while the second term is the weighted sum of the QGT for each spectral ray, as given in Eq. (12). Note that the Sjöqvist distance is calculated by taking the minimum across all possible sets of spectral phases, ensuring that it remains invariant under local gauge transformations of the form $U\left(R\right)=\text{diag}\left(e^{i\chi_{0}\left(R\right)},\ldots ,e^{i\chi_{N-1}\left(R\right)}\right)\in {\text{U}}^{N}\left(1\right)$. Indeed, this distance measures the real quantum distance between ‘adjacent’ mixed states in terms of spectral rays. Consequently, $Q_{\mu \nu }^{\text{S}}$ also remains invariant under this local U${}^{N}\left(1\right)$ transformation. Since it captures the local properties of quantum states, it is also referred to as the U${}^{N}\left(1\right)$ QGT.

### 3.2 Mathematical foundation for U${}^{N}\left(1\right)$ QGT

#### 3.2.1 Purification of density matrix

Similarly, the formalism of the U${}^{N}\left(1\right)$ QGT can also be systematically derived using the language of fiber bundles. The key point is to identify the counterpart of a pure-state wavefunction in the context of mixed states. This can be accomplished by decomposing a N-dimensional density matrix as $\rho =WW^{†}$, where W is referred to as the purification of ρ and serves a role analogous to that of the wavefunction. Conversely, the polar decomposition of W is $W=\sqrt{\rho }U$, where U∈U(N) represents the phase factor of W. It is important to note that this decomposition is unique only when ρ is full-rank. Clearly, U generalizes the U(1) phase factor $e^{i\chi }$ from pure states to mixed states. Furthermore, W also has a pure-state-like representation, the purified state |W〉. If ρ is diagonalized as $\rho =\sum_{n}\lambda_{n}\left|n\rangle \langle n\right|$, then $W=\sum_{n}\sqrt{\lambda_{n}}|n\rangle \langle n|U$, and the purified state is $|W\rangle =\sum_{n}\sqrt{\lambda_{n}}|n\rangle ⊗U^{T}|n\rangle$. In other words, |W〉 is obtained by formally applying the transpose operation to 〈n|U in W. Finally, the Hilbert-Schmidt inner product between two purifications/purified states is defined as: $\langle W_{1},W_{2}\rangle =\langle W_{1}|W_{2}\rangle =\text{Tr}\left(W_{1}^{†}W_{2}\right)$, which introduces a norm ${∥W∥}^{2}=\langle W,W\rangle$. Since Trρ=1, then $∥W∥=\sqrt{\text{Tr}(W^{†}W)}=\sqrt{\text{Tr}(WW^{†})}=1$.

It is known that the density matrix is not in one-to-one correspondence with mixed states, as stated by Schrödinger’s mixture theorem[43]. However, the density matrix is sufficient to predict any physical measurement in its associated mixed state. For an observable O, its expectation value in this mixed state is determined by 〈O〉=Tr(ρO). Thus, two different density matrices, $\rho_{1}$ and $\rho_{2}$, correspond to two physically inequivalent mixed states since there must exist at least one observable $\tilde{O}$ such that $\text{Tr}\left(\rho_{1}\tilde{O}\right)\neq \text{Tr}\left(\rho_{2}\tilde{O}\right)$. A density matrix can have infinitely many purifications, all of which correspond to a single physically equivalent mixed state. Let ρ and its purification W continuously depend on the parameter R. The distance between nearby purifications with respect to variations of R is given by

(17) $$\begin{array}{cc} ds^{2} & =∥W\left(R+dR\right)\rangle -{\left|W\left(R\right)\rangle \right|}^{2}\\ & =\langle \partial_{\mu }W|\partial_{\nu }W\rangle dR^{\mu }dR^{\nu }=\text{Tr}\left(\partial_{\mu }W^{†}\partial_{\nu }W\right)dR^{\mu }dR^{\nu }. \end{array}$$

Similar to Eq. (1), this is the ‘raw distance’ between mixed states. For comparison, the Sjöqvist distance in the previous section is invariant under local ${\text{U}}^{N}\left(1\right)$ gauge transformations, which allows it to be recognized as a proper distance between certain types of inequivalent mixed states. To obtain the Sjöqvist distance by eliminating extra gauge redundancies from the raw distance, a systematic description based on fiber bundles is required, analogous to the approach for pure states.

#### 3.2.2 Fibre bundle description

To construct a fibre bundle description, we first denote $D_{N}^{N}$ as the space spanned by all full-rank density matrices of dimension N, i.e., $\forall \rho \in D_{N}^{N}$, rank(ρ)=N. Mathematically, $D_{N}^{N}$ is a manifold equipped with a Riemannian metric[43], and it can also be referred to as the phase space of mixed states [39]. To generate the Sjöqvist distance, we select a special purification of $\rho =\sum_{n}\lambda_{n}\left|n\rangle \langle n\right|$, given by

(18) $$\begin{array}{c} W=\sum_{n}\sqrt{\lambda_{n}}|n\rangle \langle n_{0}|e^{i\theta_{n}}. \end{array}$$

Here, $\{|n_{0}\rangle \}{}_{n=0}^{N-1}$ is a set of fixed states, and their specific selection will be detailed in the following discussion. Comparing with $W=\sqrt{\rho }U$, the phase factor is given by

(19) $$\begin{array}{c} U=\sum_{n}e^{i\theta_{n}}|n\rangle \langle n_{0}|∼\left(\begin{array}{ccc} e^{i\theta_{0}} & & \\ & \ddots & \\ & & e^{i\theta_{N-1}} \end{array}\right). \end{array}$$

Thus, the set of all phase factors of a single W is isomorphic to U${}^{N}\left(1\right)$. All purifications given in Eq. (18) form the total space $S_{N}=\{W|W=\sqrt{\rho }U,\rho \in D_{N}^{N},U\in {\text{U}}^{N}\left(1\right),\;\text{and}\;∥W∥=1\}$, and the fibre space at a point ρ is $F_{\rho }∼{\text{U}}^{N}\left(1\right)$. The corresponding fibration is $S_{N}/{\text{U}}^{N}\left(1\right)=D_{N}^{N}$. Similar to the previous approach, we assume that ρ continuously depends on the parameter R, i.e. $\rho \left(R\right)=\sum_{n}\lambda_{n}\left(R\right)|n\left(R\right)\rangle \langle n\left(R\right)|$. In this context, R serves as the local coordinate of $D_{N}^{N}$. We select a fixed point $R_{0}$ and define $|n_{0}\rangle =|n\left(R_{0}\right)\rangle$. Next, we introduce the local gauge transformation $U\left(R\right)=\sum_{n}|n_{0}\rangle \langle n_{0}|e^{i\chi_{n}\left(R\right)}$, which also form a U${}^{N}\left(1\right)$ group. Let $W\in F_{\rho }$. Under the right action $W^{\prime }=WU$, the gauge transformed purification $W^{\prime }$ remains in the same fibre space $F_{\rho }$. This indicates that all U span the structure group U${}^{N}\left(1\right)$, which is isomorphic to $F_{\rho }$. Consequently, we can construct a U${}^{N}\left(1\right)$ principal bundle. The canonical projection is defined as $\pi :S_{N}\to D_{N}^{N}$ such that $\pi \left(W\right)=WW^{†}=\rho$. Conversely, the smooth map $\sigma \left(\rho \left(R\right)\right)=\sum_{n}\sqrt{\lambda_{n}\left(R\right)}|n\left(R\right)\rangle \langle n_{0}|e^{i\theta_{n}\left(R\right)}$ defines a section. Note that there always exists a global section $\sigma \left(\rho \right)=\sqrt{\rho }$, which means the phase factor is trivial. Accordingly, this principal bundle is topologically trivial, distinguishing it from the case of pure states.

The associated gauge connection of this principal bundle is a ${\underset{︸}{\text{u}(1)⊕\text{u}(1)⊕\cdots \text{u}(1)}}_{N}$-valued 1-form. In this context, after undergoing a cyclic parallel transport, the initial and final fibers will differ by a U${}^{N}\left(1\right)$-valued holonomy. Notably, we will demonstrate that a subgroup of this holonomy, specifically the U(1) group, is better suited for understanding the local geometric properties in this scenario. And the corresponding gauge connection is a u(1)-valued 1-form. Therefore, to explicitly match the minimization condition (14), we introduce this type of connection on $S_{N}$: $\omega =\langle W|d|W\rangle =\text{Tr}\left(W^{†}dW\right)$, instead of a u(N)-valued connection. Here the dependence on R is compressed for convenience. Using the fact that $\sum_{n}\sqrt{\lambda_{n}}d\sqrt{\lambda_{n}}=d\sum_{n}\lambda_{n}=0$, it can be found that

(20) $$\begin{array}{c} \omega =\sum_{n}\lambda_{n}\left(id\theta_{n}+A_{n}\right) \end{array}$$

where $A_{n}=\langle n|dn\rangle$ is the Berry connection for the n-the level |n〉. Similarly, the first and second terms on the right-hand-side represent the associated connections on the fibre space and the base manifold respectively. This formulation is a direct generalization of the pure-state case. Under the local gauge transformation $W^{\prime }=WU$, ω transforms as $\omega^{\prime }=\omega +i\sum_{n}\lambda_{n}d\chi_{n}$, thereby it is indeed a U(1) connection. Moreover, ω is the Ehresmann connection on $S_{N}$. Consider a loop R(t) (0≤t≤τ) in M, which satisfies $R\left(0\right)=R\left(\tau \right)=R_{0}$. It also induces a loop γ(t)=ρ(t)≡ρ(R(t)) in $D_{N}^{N}$. The lift of γ(t) is a curve in $S_{N}$: $\tilde{\gamma }\left(t\right)=\sigma \left(\rho \left(t\right)\right)=W\left(t\right)\equiv W\left(R\left(t\right)\right)$, which is not necessarily closed. Denote the tangent vectors of γ and $\tilde{\gamma }$ by X and $\tilde{X}$, respectively. If $\tilde{\gamma }$ is a horizontal lift of γ, then $\omega (\tilde{X})=0$, which implies

(21) $$\text{Tr}(W^{†}\dot{W})=\sum_{n}\lambda_{n}\left(i{\dot{\theta }}_{n}\left(t\right)+A_{n}\left(\tilde{X}\right)\right)=\sum_{n}\lambda_{n}\left(i{\dot{\theta }}_{n}\left(t\right)+\langle n\left(t\right)|\frac{d}{dt}|n\left(t\right)\rangle \right)=0.$$

If this condition is met, W(t) is said to be parallel transported along R(t). Comparing with Eq. (14), it can be found that the minimization condition for Sjöqvist distance is a sufficient condition for this parallel-transport condition. Furthermore, the pullback of ω by σ defines a U(1) connection on the base manifold:

(22) $$\begin{array}{c} A_{D_{N}^{N}}=\sigma^{*}\omega =\sum_{n}\lambda_{n}A_{n}. \end{array}$$

In context of the U${}^{N}\left(1\right)$ principal bundle, Eq. (17) in fact represents the local distance on the total space $S_{N}$, i.e. the ‘raw distance’ $ds^{2}\left(S_{N}\right)$ between adjacent mixed states. A straightforward evaluation shows that

(23) $$ds^{2}\left(S_{N}\right)=\sum_{n}\{{\left(d\sqrt{\lambda_{n}}\right)}^{2}+\lambda_{n}[\langle dn|dn\rangle +{\left(d\theta_{n}-iA_{n}\right)}^{2}+{\left(A_{n}\right)}^{2}]\},$$

where $A_{n\mu }=\langle n|\partial_{\mu }n\rangle =-\langle \partial_{\mu }n|n\rangle$ is the nth component of $A_{n}$, and the second line is expressed in terms of differential forms. Obviously, the raw distance is minimized when

(24) $$\begin{array}{c} \partial_{\mu }\theta_{n}-iA_{n\mu }=0,\;\;\text{or}\;\;d\theta_{n}-iA_{n}=0, \end{array}$$

which exactly agrees with Eq. (14), the minimization condition of the Sjöqvist distance. Similar to Eq. (10), the raw distance can also be decomposed as

(25) $$\begin{array}{c} ds^{2}\left(S_{N}\right)=ds^{2}\left(D_{N}^{N}\right)+\sum_{n}\lambda_{n}{\left(d\theta_{n}-iA_{n}\right)}^{2}. \end{array}$$

**Fig. 1:**
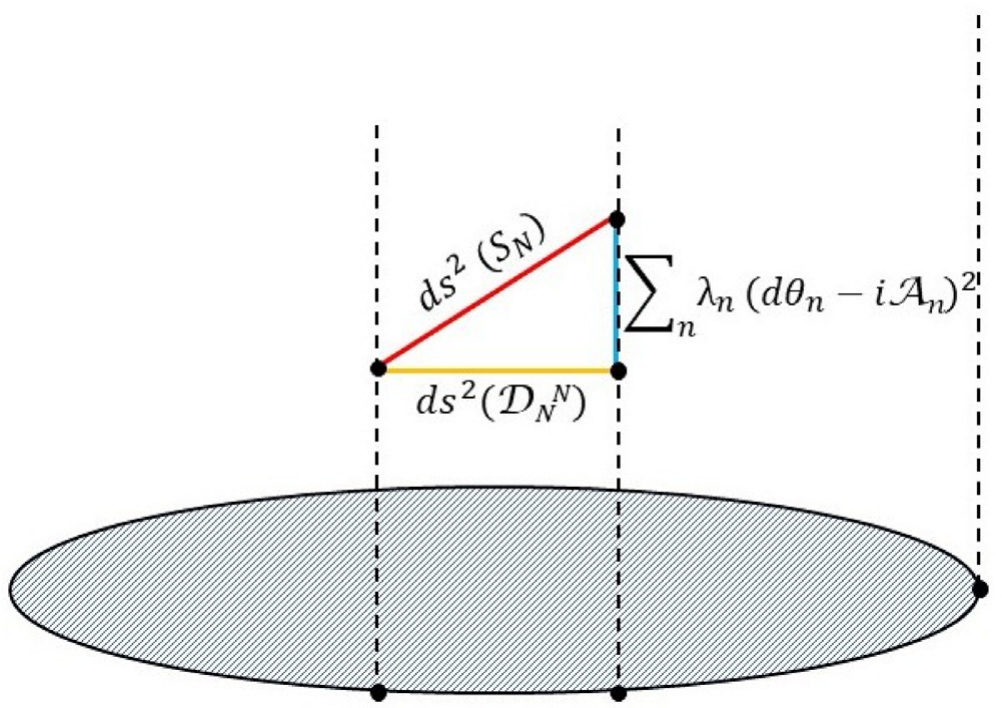
**A schematic illustrating the geometric relationships among the distances in the total space, fiber space, and base manifold**.

Here

$$ds^{2}\left(D_{N}^{N}\right)=\sum_{n}\left\{{\left(d\sqrt{\lambda_{n}}\right)}^{2}+\lambda_{n}[\langle dn|dn\rangle +{\left(A_{n}\right)}^{2}]\right\}=\sum_{n}\left[\frac{{\left(d\lambda_{n}\right)}^{2}}{4\lambda_{n}}+\lambda_{n}\langle dn|(1-|n\rangle \langle n|)|dn\rangle \right]$$

is the distance on the base manifold $D_{N}^{N}$, which is also the Sjöqvist distance by comparing with Eq. (15). The second term $\sum_{n}\lambda_{n}{\left(d\theta_{n}-iA_{n}\right)}^{2}$ represents the local distance on the fibre space. This decomposition is entirely consistent with the fibration $S_{N}/{\text{U}}^{N}\left(1\right)=D_{N}^{N}$. To illustrate this relationship, we present a schematic in Fig. 1 that depicts the geometric connections among these distances. When the total distance $ds^{2}\left(S_{N}\right)$ is minimized to $ds^{2}\left(D_{N}^{N}\right)$, the associated purification must undergoes a parallel transport. However, this does not hold conversely, as the parallel transport condition is merely a necessary implication of the minimization condition (14). The situation differs from that of pure states. This is because the U(1) connection ω does not fully match the U${}^{N}\left(1\right)$ fibre space. As previously mentioned, $ds^{2}\left(D_{N}^{N}\right)$ or the Sjöqvist distance can be further separated into $ds^{2}\left(D_{N}^{N}\right)=ds_{\text{FR}}^{2}+\sum_{n}\lambda_{n}ds_{\text{FS}n}^{2}$, where $ds_{\text{FR}}^{2}=\sum_{n}\frac{{\left(d\lambda_{n}\right)}^{2}}{4\lambda_{n}}$ is the Fisher-Rao distance, and $ds_{\text{FS}n}^{2}=\langle dn|(1-|n\rangle \langle n|)|dn\rangle$ is the Fubini-Study distance for the nth level.

Since all contribution from the phase factors has been eliminated by the projection π, $ds^{2}\left(D_{N}^{N}\right)$ or the Sjöqvist distance must remain invariant under any local U${}^{N}\left(1\right)$ gauge transformation. The corresponding metric is given by Eq. (16), which is also U${}^{N}\left(1\right)$-invariant. As we stated before, it is also referred to as the U${}^{N}\left(1\right)$ QGT. According to the decomposition of distances, the U${}^{N}\left(1\right)$ QGT can also be decomposed into

(26) $$\begin{array}{c} Q_{\mu \nu }^{\text{S}}=g_{\mu \nu }^{\text{FR}}+g_{\mu \nu }^{\text{FS}}-i\Omega_{\mu \nu }, \end{array}$$

where $g_{\mu \nu }^{\text{FR}}=\sum_{n}\frac{\partial_{\mu }\lambda_{n}\partial_{\nu }\lambda_{n}}{4\lambda_{n}}$ is the Fisher-Rao metric, $g_{\mu \nu }^{\text{FS}}=\sum_{n}\lambda_{n}g_{n\mu \nu }^{\text{FS}}=\sum_{n}\lambda_{n}\left(\text{Re}\langle \partial_{\mu }n|\partial_{\nu }n\rangle +A_{n\mu }A_{n\nu }\right)$ is the weighted sum of the Fubini-Study metrics for each spectral ray, and the negative imaginary part

(27) $$\begin{array}{cc} \Omega_{\mu \nu } & =\frac{i}{2}\sum_{n}\lambda_{n}\left(\langle \partial_{\mu }n|\partial_{\nu }n\rangle -\langle \partial_{\nu }n|\partial_{\mu }n\rangle \right)\\ & \equiv \frac{i}{2}\sum_{n}\lambda_{n}F_{n\mu \nu } \end{array}$$

is half of the weighted sum of the Berry curvature for each spectral ray. Comparing to discussions in Section 2.2, the formalism developed here indeed serves as a suitable generalization to that of pure states. We summarize our main findings by comparing the key points between pure and mixed states in Table 1, where the results in the left column come from our previous work in Ref. [39].

**Table 1:** **A comparison between the geometries of pure and mixed states**.

	Pure state	Mixed state
Total space	$S^{2N-1}$	$S_{N}$
Phase space	$CP^{N-1}$	$D_{N}^{N}$
Fibration	$S^{2N-1}/\text{U}\left(1\right)=CP^{N-1}$	$S_{N}/{\text{U}}^{N}\left(1\right)=D_{N}^{N}$
Connection	Berry connection A	Weighted sum of Berry connection $\sum_{n}\lambda_{n}A_{n}$
Raw distance	$ds^{2}\left(S^{2N-1}\right)$	$ds^{2}\left(S_{N}\right)$
Gauge-invariant distance	$ds^{2}\left(CP^{N-1}\right)$	$ds^{2}\left(D_{N}^{N}\right)$
Relations between distances	$ds^{2}\left(S^{2N-1}\right)=ds^{2}\left(CP^{N-1}\right)+{\left(d\theta -iA\right)}^{2}$	$ds^{2}\left(S_{N}\right)=ds^{2}\left(D_{N}^{N}\right)+\sum_{n}\lambda_{n}{\left(d\theta_{n}-iA_{n}\right)}^{2}$
Raw metric	$\langle \partial_{\mu }\tilde{\psi }|\partial_{\nu }\tilde{\psi }\rangle$	$\langle \partial_{\mu }W|\partial_{\nu }W\rangle$
Real part of QGT	Re$\langle \partial_{\mu }\psi |\partial_{\nu }\psi \rangle +A_{\mu }A_{\nu }$ (Fubini-Study)	$\sum_{n}\left[\frac{\partial_{\mu }\lambda_{n}\partial_{\nu }\lambda_{n}}{4\lambda_{n}}+\lambda_{n}\left(\text{Re}\langle \partial_{\mu }n|\partial_{\nu }n\rangle +A_{n\mu }A_{n\nu }\right)\right]$
Imaginary part of QGT	Berry curvature $\frac{i}{2}F$	Weighted sum of Berry curvature $\frac{i}{2}\sum_{n}\lambda_{n}F_{n}$

Since $\lambda_{n}$ generically depends on R, $\Omega_{\mu \nu }$ is not necessarily the gauge potential associated with $A_{D_{N}^{N}}$. In other words, $\Omega_{\mu \nu }=\frac{i}{2}\left[\partial_{\mu }\left(\sum_{n}\lambda_{n}A_{n\nu }\right)-\partial_{\nu }\left(\sum_{n}\lambda_{n}A_{n\mu }\right)\right]$ is not true in general. Nevertheless, $\Omega_{\mu \nu }$ is a gauge-invariant quantity. We introduce the 2-form $\Omega =\frac{1}{2}\Omega_{\mu \nu }dR^{\mu }\wedge dR^{\nu }=\frac{i}{2}\sum_{n}\lambda_{n}F_{n}$, where $F_{n}=\frac{1}{2}F_{n\mu \nu }dR^{\mu }\wedge dR^{\nu }$ is the Berry curvature 2-form for the nth level. The integral of Ω on a parameter surface Σ is also gauge-invariant. Let C be the boundary of Σ. We further define a gauge-invariant quantity

(28) $$\begin{array}{c} \theta_{g}\left(C\right)=\int_{\Sigma }\Omega =\frac{i}{2}\sum_{n}\int_{\Sigma }\lambda_{n}F_{n}. \end{array}$$

If all $\lambda_{n}$ are constant on Σ, then we have

(29) $$\begin{array}{c} \theta_{g}\left(C\right)=\frac{i}{2}\sum_{n}\lambda_{n}\int_{\Sigma }F_{n}=\frac{1}{2}\sum_{n}\lambda_{n}\theta_{\text{B}n}\left(C\right), \end{array}$$

which is half the weighted sum of all Berry phases.

#### 3.2.3 Geometrical implication of the imaginary part

The imaginary part of the pure-state QGT is proportional to the U(1) Berry curvature, establishing a direct connection with the topological properties of the pure-state manifold, or more accurately, the U(1) principal bundle. In our previous work (Ref. [39])), we extended the concept of QGT to mixed states by imposing the U(N) gauge-invariant condition, which is a stringent requirement. This was accomplished by constructing a U(N) principal bundle and utilizing the Uhlmann connection. However, this bundle is topologically trivial, and the imaginary part of QGT vanishes in typical physical processes.

But what about the U${}^{N}\left(1\right)$ QGT we introduced here? It is noteworthy that the U${}^{N}\left(1\right)$ principal bundle is also topologically trivial owing to the existence of a global section, as previously mentioned. To describe the parallel transport of the U${}^{N}\left(1\right)$ fiber, it is necessary to introduce a U${}^{N}\left(1\right)$-valued gauge connection. However, such a connection is challenging to reconcile with both the gauge transformation conditions and the condition of minimizing distance. Moreover, the associated gauge curvature is necessarily trivial because the U${}^{N}\left(1\right)$ principal bundle is topologically trivial. Consequently, the derived QGT may also lack the imaginary part, just as the U(N) QGT in Ref. [39].

Instead, we consider a simpler U(1) (⊂ U${}^{N}\left(1\right)$) gauge connection, which geometrically implies that after performing periodic parallel transport of the U${}^{N}\left(1\right)$ fiber, the initial and final fibers differ by a U(1) phase. The resulting QGT contains both a real and an imaginary part: the real part accurately characterizes the local distance on the mixed-state manifold, while the imaginary part does not reflect the global topological properties of the U${}^{N}\left(1\right)$ principal bundle. Instead, it represents the geometric properties arising from U(1) parallel transport of the U${}^{N}\left(1\right)$ fiber.

### 3.3 Fundamental inequalities about the U${}^{N}\left(1\right)$ QGT

It is known that the pure-state QGT, as described in Eq. (5), satisfies a fundamental inequality

(30) $$\begin{array}{c} Q_{\mu \mu }Q_{\nu \nu }\geq {\left|Q_{\mu \nu }\right|}^{2}, \end{array}$$

which has intriguing applications in condensed matter physics. In two-dimensional models, it establishes an inequality relationship between the first Chern number and the volume of the quantum states [8], [44]. Here we show that the U${}^{N}\left(1\right)$ QGT for mixed states holds the same result:

(31) $$\begin{array}{c} Q_{\mu \mu }^{\text{S}}Q_{\nu \nu }^{\text{S}}\geq {\left|Q_{\mu \nu }^{\text{S}}\right|}^{2}. \end{array}$$

Let $g_{n\mu \nu }^{\text{FR}}=\frac{\partial_{\mu }\lambda_{n}\partial_{\nu }\lambda_{n}}{4\lambda_{n}}$ and $Q_{\mu \nu }^{n}=g_{n\mu \nu }^{\text{FS}}-\frac{i}{2}F_{n\mu \nu }$ represent the Fisher-Rao metric and the pure-state QGT for the nth eigenstate of ρ, respectively. Then, $Q_{\mu \nu }^{\text{S}}=\sum_{n}\left(g_{n\mu \nu }^{\text{FR}}+\lambda_{n}Q_{\mu \nu }^{n}\right)$. We first prove the follow inequalities:

(32) $$\begin{array}{cc} \sum_{n}g_{n\mu \mu }^{\text{FR}}\sum_{m}g_{m\nu \nu }^{\text{FR}} & \geq |\sum_{n}g_{n\mu \nu }^{\text{FR}}{|}^{2}, \end{array}$$

(33) $$\begin{array}{cc} \sum_{n}\lambda_{n}Q_{\mu \mu }^{n}\sum_{m}\lambda_{m}Q_{\nu \nu }^{m} & \geq |\sum_{n}\lambda_{n}Q_{\mu \nu }^{n}{|}^{2}. \end{array}$$

Noting $g_{n\mu \mu }^{\text{FR}}={\left(\frac{\partial_{\mu }\lambda_{n}}{2\sqrt{\lambda_{n}}}\right)}^{2}$ and applying the Cauchy-Schwarz inequality

(34) $$\begin{array}{c} \sum_{mn}{\left(\frac{\partial_{\mu }\lambda_{n}}{2\sqrt{\lambda_{n}}}\right)}^{2}{\left(\frac{\partial_{\nu }\lambda_{m}}{2\sqrt{\lambda_{m}}}\right)}^{2}\geq {\left(\sum_{n}\frac{\partial_{\mu }\lambda_{n}\partial_{\nu }\lambda_{n}}{4\lambda_{n}}\right)}^{2}, \end{array}$$

we find that Inequality Eq. (32) is clearly valid. To prove Inequality Eq. (33), we introduce the projection operator $P_{n}=\left|n\rangle \langle n\right|$, leading to $Q_{\mu \nu }^{n}=\langle \partial_{\mu }n|\left(1-P_{n}\right)|\partial_{\nu }n\rangle$. Next, let

(35) $$\begin{array}{cc} |\alpha \rangle & =\left(\begin{array}{c} \sqrt{\lambda_{0}}\left(1-P_{0}\right)|\partial_{\mu }0\rangle \\ \sqrt{\lambda_{1}}\left(1-P_{1}\right)|\partial_{\mu }1\rangle \\ \vdots \\ \sqrt{\lambda_{N-1}}\left(1-P_{N-1}\right)|\partial_{\mu }\left(N-1\right)\rangle \end{array}\right),\\ |\beta \rangle & =\left(\begin{array}{c} \sqrt{\lambda_{0}}\left(1-P_{0}\right)|\partial_{\nu }0\rangle \\ \sqrt{\lambda_{1}}\left(1-P_{1}\right)|\partial_{\nu }1\rangle \\ \vdots \\ \sqrt{\lambda_{N-1}}\left(1-P_{N-1}\right)|\partial_{\nu }\left(N-1\right)\rangle \end{array}\right). \end{array}$$

Using ${\left(1-P_{n}\right)}^{2}=1-P_{n}$, we obtain $\langle \alpha |\alpha \rangle =\sum_{n}\lambda_{n}Q_{\mu \mu }^{n}$, $\langle \beta |\beta \rangle =\sum_{m}\lambda_{m}Q_{\nu \nu }^{m}$ and $\langle \alpha |\beta \rangle =\sum_{n}\lambda_{n}Q_{\mu \nu }^{n}$. By again applying the Cauchy-Schwarz inequality $\langle \alpha |\alpha \rangle \langle \beta |\beta \rangle \geq {\left|\langle \alpha |\beta \rangle \right|}^{2}$, we can deduce Inequality Eq. (33). The same proof process can also yield the following result (Taking N=1 and $\lambda_{n}=1$):

(36) $$\begin{array}{c} Q_{\mu \mu }^{n}Q_{\nu \nu }^{n}\geq {\left|Q_{\mu \nu }^{n}\right|}^{2}. \end{array}$$

The two sides of Inequality Eq. (31) are respectively expanded as

(37) $$\begin{array}{cc} Q_{\mu \mu }^{\text{S}}Q_{\nu \nu }^{\text{S}}= & \sum_{nm}\left(g_{n\mu \mu }^{\text{FR}}g_{m\nu \nu }^{\text{FR}}+\lambda_{n}Q_{\mu \mu }^{n}\lambda_{m}Q_{\nu \nu }^{m}\right)\\ + & \sum_{nm}g_{n\mu \mu }^{\text{FR}}\lambda_{m}\left(Q_{\mu \mu }^{m}+Q_{\nu \nu }^{m}\right),\\ |Q_{\mu \nu }^{\text{S}}{|}^{2}= & |\sum_{n}g_{n\mu \nu }^{\text{FR}}{|}^{2}+{\left|\sum_{n}\lambda_{n}Q_{\mu \nu }^{n}\right|}^{2}\\ + & \sum_{nm}\lambda_{m}g_{n\mu \nu }^{\text{FR}}\left(Q_{\mu \nu }^{m}+{\bar{Q}}_{\mu \nu }^{m}\right), \end{array}$$

where we have interchanged the indices n and m in the cross terms. Inequalities Eqs. (32) and (33) imply

(38) $$\begin{array}{cc} & \sum_{nm}\left(g_{n\mu \mu }^{\text{FR}}g_{m\nu \nu }^{\text{FR}}+\lambda_{n}Q_{\mu \mu }^{n}\lambda_{m}Q_{\nu \nu }^{m}\right)\\ \geq & |\sum_{n}g_{n\mu \nu }^{\text{FR}}{|}^{2}+{\left|\sum_{n}\lambda_{n}Q_{\mu \nu }^{n}\right|}^{2}. \end{array}$$

Next, applying the facts that $g_{n\mu \mu }^{\text{FR}}\geq 0$, $\lambda_{n}\geq 0$ and $g_{n\mu \mu }^{\text{FR}}g_{n\nu \nu }^{\text{FR}}={\left(g_{n\mu \nu }^{\text{FR}}\right)}^{2}$, and Inequality Eq. (36), it can be found

(39) $$\begin{array}{ccc} & & \sum_{nm}\lambda_{m}\left(g_{n\mu \mu }^{\text{FR}}Q_{\nu \nu }^{m}+g_{n\nu \nu }^{\text{FR}}Q_{\mu \mu }^{m}\right)\geq 2\sum_{nm}\lambda_{m}\sqrt{g_{n\mu \mu }^{\text{FR}}g_{n\nu \nu }^{\text{FR}}Q_{\mu \mu }^{m}Q_{\nu \nu }^{m}}\\ & & \;\;\geq \sum_{nm}g_{n\mu \nu }^{\text{FR}}\lambda_{m}2\left|Q_{\mu \nu }^{m}\right|\geq \sum_{nm}g_{n\mu \nu }^{\text{FR}}\lambda_{m}\left(Q_{\mu \nu }^{m}+{\bar{Q}}_{\mu \nu }^{m}\right). \end{array}$$

Finally, this inequality, together with Inequality Eq. (38), can jointly deduce Inequality Eq. (31). To determine the saturation condition, we observe that the equalities in Eqs. (32) and (33) hold if and only if

(40) $$\begin{array}{cc} & \frac{g_{0\mu \mu }^{\text{FR}}}{g_{0\nu \nu }^{\text{FR}}}=\cdots =\frac{g_{N-1\mu \mu }^{\text{FR}}}{g_{N-1\nu \nu }^{\text{FR}}}\Rightarrow \frac{\partial_{\mu }\lambda_{0}}{\partial_{\nu }\lambda_{0}}=\cdots =\frac{\partial_{\mu }\lambda_{N-1}}{\partial_{\nu }\lambda_{N-1}}\\ & \frac{Q_{\mu \mu }^{0}}{Q_{\nu \nu }^{0}}=\cdots =\frac{Q_{\mu \mu }^{N-1}}{Q_{\nu \nu }^{N-1}}\Rightarrow \frac{g_{0\mu \mu }^{\text{FS}}}{g_{0\nu \nu }^{\text{FS}}}=\cdots =\frac{g_{N-1\mu \mu }^{\text{FS}}}{g_{N-1\nu \nu }^{\text{FS}}}. \end{array}$$

The condition for the equality on the far left in Eq. (39) to hold is $g_{n\mu \mu }^{\text{FR}}Q_{\nu \nu }^{m}=g_{n\nu \nu }^{\text{FR}}Q_{\mu \mu }^{m}$. Together with Eq. (40), this leads to the following relation:

(41) $$\begin{array}{cc} & \frac{g_{0\mu \mu }^{\text{FR}}}{g_{0\nu \nu }^{\text{FR}}}=\cdots =\frac{g_{N-1\mu \mu }^{\text{FR}}}{g_{N-1\nu \nu }^{\text{FR}}}=\frac{g_{0\mu \mu }^{\text{FS}}}{g_{0\nu \nu }^{\text{FS}}}=\cdots =\frac{g_{N-1\mu \mu }^{\text{FS}}}{g_{N-1\nu \nu }^{\text{FS}}}. \end{array}$$

This implies that the ratio between the different diagonal terms of the Fisher-Rao metric and the Fubini-Study metric for each eigenlevel is a constant. If we define 2k vectors as follows: $g_{\mu }^{\text{FR}}=\left(g_{0\mu \mu }^{\text{FR}},\ldots ,g_{N-1\mu \mu }^{\text{FR}}\right)$, $g_{\mu }^{\text{FS}}=\left(g_{0\mu \mu }^{\text{FS}},\ldots ,g_{N-1\mu \mu }^{\text{FS}}\right)$, μ=1,2,…,k, these vectors are all linearly dependent. Moreover, the condition for the equality on the far right in Eq. (39) to hold is that all cross terms $Q_{\mu \nu }^{n}$ are real.

In Ref. [39], we pointed out that the real part of the U(N) QGT is the Bures metric. In fact, Bures metric also respect a similar inequality. We present the proof in the Appendix A.

If the parameter space is two dimensional, i.e. $R=(R^{1},R^{2})$, the U${}^{N}\left(1\right)$ QGT can be expressed as a 2×2 matrix and the nontrivial result of Inequality Eq. (31) is

(42) $$\begin{array}{c} Q_{11}^{\text{S}}Q_{22}^{\text{S}}\geq {\left|Q_{12}^{\text{S}}\right|}^{2}. \end{array}$$

Let $g_{\mu \nu }^{\text{S}}$ be the real part of $Q_{\mu \nu }^{\text{S}}$. For convenience, we also introduce $F_{\mu \nu }=-2\text{Im}Q_{\mu \nu }^{\text{S}}=\sum_{n}\lambda_{n}F_{n\mu \nu }$. The U${}^{N}\left(1\right)$ QGT can be written as

(43) $$\begin{array}{c} Q^{\text{S}}=\left(\begin{array}{cc} g_{11}^{\text{s}} & g_{12}^{\text{S}}-\frac{i}{2}F_{12}\\ g_{21}^{\text{S}}-\frac{i}{2}F_{21} & g_{22}^{\text{S}} \end{array}\right)=g^{\text{S}}-\frac{iF_{12}}{2}\left(\begin{array}{cc} 0 & 1\\ -1 & 0 \end{array}\right). \end{array}$$

The Inequality Eq. (42) implies

(44) $$\begin{array}{cc} & \det\left(g^{\text{S}}\right)=g_{11}^{\text{S}}g_{22}^{\text{S}}-{\left(g_{12}^{\text{S}}\right)}^{2}\geq \frac{1}{4}{\left|F_{12}\right|}^{2},\\ & \;\;\;\text{or}\;\sqrt{\det(g^{\text{S}})}\geq \frac{|F_{12}|}{2}. \end{array}$$

This is a direct generalization to the results for pure-states [8]. Define

(45) $$\begin{array}{c} V_{g}^{\text{S}}=\int d^{2}R\sqrt{\det(g^{\text{S}})}, \end{array}$$

which is the quantum volume of the parameter space. Inequality Eq. (44) yields

(46) $$\begin{array}{c} V_{g}^{\text{S}}\geq \int d^{2}R\frac{|F_{12}|}{2}\geq \left|\frac{i}{2}\int d^{2}R\sum_{n}\lambda_{n}F_{n12}\right|=\left|\theta_{g}\right|. \end{array}$$

This builds a relation between $V_{g}^{\text{S}}$ and $\theta_{g}$, providing a lower bound for the parameter volume $V_{g}^{\text{S}}$.

### 3.4 Examples of U${}^{N}\left(1\right)$ QGT

#### 3.4.1 Bosonic coherent state

We first calculate the U${}^{N}\left(1\right)$ QGT of bosonic coherent states, which can be constructed from bosonic harmonic oscillators [45], [46] and has been experimentally applied in quantum optics [47], [48], [49]. The Hamiltonian of a single harmonic oscillator is given by $\hat{H}=\hbar \omega \left(a^{†}a+\frac{1}{2}\right)$, where a and $a^{†}$ are the annihilation and creation operators, respectively, satisfying $[a,a^{†}]=1$. The energy levels of system satisfy $\hat{H}|n\rangle =\hbar \omega \left(n+\frac{1}{2}\right)|n\rangle$ with n=0,1,2,…. The coherent state is defined by operating the translation operator on the ground state: $|z\rangle =D\left(z\right)|0\rangle \equiv e^{za^{†}-\bar{z}a}|0\rangle$. This can be generalized to exited state: |n,z〉=D(z)|n〉, n≥1. The density matrix is obtained via the transformation D(z): $\rho \left(z\right)=\frac{1}{Z}e^{-\beta \hat{H}\left(z\right)}=D\left(z\right)\rho \left(0\right)D^{†}\left(z\right)$, where $\rho \left(0\right)=\frac{1}{Z}e^{-\beta \hat{H}}$ with β being the inverse temperature. Since ρ(0) shares the same eigenstates with $\hat{H}$, the eigenstates of ρ(z) are |n,z〉 with n=0,1,2,…. Note the eigenvalues $\lambda_{n}=\frac{1}{Z}e^{-\beta \hbar \omega (n+\frac{1}{2})}$ are constants, then the Sjöqvist distance is

(47) $$ds_{\text{S}}^{2}=\sum_{n}\lambda_{n}d\langle n,z|(1-|n,z\rangle \langle n,z|)d|n,z\rangle =\sum_{n}\lambda_{n}[\langle n|dD^{†}\left(z\right)dD\left(z\right)|n\rangle -\langle n|dD^{†}\left(z\right)D\left(z\right)|n\rangle \langle n|D^{†}\left(z\right)dD\left(z\right)|n\rangle ].$$

Applying

(48) $$\langle n|dD^{†}\left(z\right)dD\left(z\right)|n\rangle =\left(2n+1+\frac{{\left|z\right|}^{2}}{2}\right)dzd\bar{z}-\frac{z^{2}}{4}d{\bar{z}}^{2}-\frac{{\bar{z}}^{2}}{4}dz^{2},$$

a straightforward evaluation shows

$$ds_{\text{S}}^{2}=\sum_{n}\left(2n+1\right)\lambda_{n}dzd\bar{z}=\text{coth}\left(\frac{\beta \hbar \omega }{2}\right)dzd\bar{z}=\text{coth}\left(\frac{\beta \hbar \omega }{2}\right)\left(dx^{2}+dy^{2}\right).$$

In the zero temperature limit, it reduces to the Fubini-Study distance [1]: $ds_{\text{S}}^{2}=dx^{2}+dy^{2}$. This is reasonable since the contributions from all excited states fade away as β→+∞. At the infinite temperature,

(49) $$\begin{array}{c} \lim_{\beta \to 0}ds_{\text{S}}^{2}=\left(dx^{2}+dy^{2}\right)\lim_{\beta \to 0}\text{coth}\left(\frac{\beta \hbar \omega }{2}\right)\to +\infty . \end{array}$$

Even the Sjöqvist distance between adjacent coherent states diverges. This may be due to the existence of an infinite number of energy levels, each of which carries the same weight at infinite temperature, resulting in a divergent total contribution from the Fubini-Study distances of all energy levels.

The real part of the U${}^{N}\left(1\right)$ QGT can be immediately obtained from the expression of $ds_{\text{S}}^{2}$. According to Eq. (27), in terms of 2-form, the negative imaginary part is

(50) $$\Omega =\frac{i}{2}\sum_{n}\lambda_{n}d\langle n,z|\wedge d|n,z\rangle =-\frac{i}{2}dz\wedge d\bar{z}=-dx\wedge dy.$$

#### 3.4.2 Fermionic coherent state

The Hamiltonian of a fermionic harmonic oscillator is $\hat{H}=\frac{\hbar \omega }{2}\left[b^{†},b\right]=\hbar \omega \left(b^{†}b-\frac{1}{2}\right)$, where the fermionic operators b and $b^{†}$ satisfy the algebra $\{b,b^{†}\}=1$ [46], [50]. The fermionic coherent state was first introduced to physics Julian Schwinger in 1953 [51] as a means to construct a functional formulation of quantum field theory. Its theoretical framework gradually developed and matured in the following years [52], [53], [54], [55]. Similar to its bosonic counterpart, the fermionic coherent state is also constructed by applying a translation to the vacuum: $|\xi \rangle =D\left(\xi \right)|0\rangle \equiv e^{b^{†}\xi -\bar{\xi }b}|0\rangle$, where ξ is a Grassmann number and anticommutes with any fermionic operator. The corresponding density matrix is

(51) $$\begin{array}{c} \rho \left(\xi \right)=\frac{1}{Z}e^{-\beta D\left(\xi \right)\hat{H}D^{†}\left(\xi \right)}=D\left(\xi \right)\rho \left(0\right)D^{†}\left(\xi \right), \end{array}$$

where $\rho \left(0\right)=\frac{e^{-\beta \hat{H}}}{Z}$ with the partition function $Z=e^{\frac{1}{2}\beta \hbar \omega }+e^{-\frac{1}{2}\beta \hbar \omega }=2\cosh\frac{\beta \hbar \omega }{2}$. Using the algebra ${\left(b^{†}b\right)}^{2}=b^{†}b$, it can be found that

(52) $$\begin{array}{cc} \rho (\xi ) & =\frac{1}{1+e^{-\beta \hbar \omega }}-\tanh\left(\frac{\beta \hbar \omega }{2}\right)\left(b^{†}-\bar{\xi }\right)\left(b-\xi \right). \end{array}$$

The eigenvalues of ρ(ξ) are independent of ξ, then the Sjöqvist distance is

$$\begin{array}{cc} ds_{\text{S}}^{2}= & \sum_{n=0,1}\lambda_{n}\langle n|dD^{†}\left(\xi \right)[1-D\left(\xi \right)|n\rangle \langle n|D^{†}\left(\xi \right)]dD\left(\xi \right)|n\rangle . \end{array}$$

Applying

$$\begin{array}{cc} dD(\xi )= & \left(b^{†}-\frac{1}{2}\bar{\xi }\right)D\left(\xi \right)d\xi +D\left(\xi \right)\left(b+\frac{1}{2}\xi \right)d\bar{\xi }\\ dD^{†}\left(\xi \right)= & -D^{†}\left(\xi \right)\left(b-\frac{1}{2}\xi \right)d\bar{\xi }-\left(b^{†}+\frac{1}{2}\bar{\xi }\right)D^{†}\left(\xi \right)d\xi , \end{array}$$

we obtain

(53) $$\begin{array}{c} ds_{\text{S}}^{2}=\lambda_{0}d\xi d\bar{\xi }-\lambda_{1}d\bar{\xi }d\xi =\tanh\left(\frac{\beta \hbar \omega }{2}\right)d\bar{\xi }d\xi . \end{array}$$

In the zero temperature limit, it reduces to $ds_{\text{S}}^{2}=d\bar{\xi }d\xi$. At the infinite temperature, $ds_{\text{S}}^{2}\to 0$, which sharply contrasts with the results for bosonic coherent states. In terms of 2-form, the negative imaginary part is

(54) $$\begin{array}{cc} \Omega = & \frac{i}{2}\sum_{n}\lambda_{n}\langle n|dD^{†}\left(\xi \right)\wedge dD\left(\xi \right)|n\rangle =d\xi \wedge d\bar{\xi }. \end{array}$$

#### 3.4.3 Superconductor

The QGTs in the two previous models have no cross terms, ensuring that Inequality Eq. (31) is naturally satisfied. Here, we explore a more complex example: the continuum model of superconductivity, specifically focusing on the conventional 3D s-wavel BCS superconductors. With the help of the Nambu spinor $\Psi_{k}={\left(\psi_{k↑},\psi_{-k↓}^{†}\right)}^{T}$, representing two-component fermions with spins ↑ and ↓, the mean-field Hamiltonian for a three-dimensional superconductor is expressed as $\hat{H}=\sum_{k}\Psi_{k}^{†}H\left(k\right)\Psi_{k}$, where $H\left(k\right)=d_{1}\sigma_{1}+d_{3}\sigma_{3}$ with $d_{1}=-\Delta$ being the (negative) energy gap and $d_{3}=\xi_{k}=\frac{k^{2}}{2m}-\mu$. Here, m denotes the electron mass, μ is the chemical potential, and the QGT will be evaluated with respect to the continuous parameter $k={\left(k_{x},k_{y},k_{z}\right)}^{T}$. The gap is determined by the equation $\Delta =U\sum_{k}\langle \psi_{k↑}\psi_{-k↓}\rangle$, where U represents the pairing coupling constant. This model has two energy bands, described by $\pm E_{k}\equiv \pm d\left(k\right)=\pm \sqrt{\Delta^{2}+\xi_{k}^{2}}$. The associated energy levels are

(55) $$\begin{array}{cc} & |u_{\pm }\rangle =\frac{1}{\sqrt{2d\left(d\pm d_{3}\right)}}\left(\begin{array}{c} d\pm d_{3}\\ \pm d_{1} \end{array}\right). \end{array}$$

The state of the system is determined by solving the number equation

(56) $$n=\sum_{k}\left[1-\frac{\xi_{k}}{E_{k}}(1-2f\left(E_{k}\right))\right]$$

and the gap equation

(57) $$\frac{1}{U}+\sum_{k}\frac{1-2f(E_{k})}{2E_{k}}=0$$

simultaneously. Here n represents the number density, and $f\left(x\right)={\left(e^{\frac{x}{T}}+1\right)}^{-1}$ is the Fermi-Dirac distribution function. The superconducting transition temperature, $T_{c}$, is defined by the conditions Δ>0 for $T<T_{c}$ and Δ=0 for $T>T_{c}$.

The thermal-equilibrium density matrix is found to be $\rho_{k}=\frac{1}{2}\left[1-\tanh\left(\beta E_{k}\right)\hat{d}\left(k\right)\cdot \sigma \right]$ where $\hat{d}=d/d$ with $d={\left(d_{1},0,d_{3}\right)}^{T}$. According to Eq. (26), a straightforward evaluation shows that the QGT in this model is expressed as $Q_{ij}^{\text{S}}=g_{ij}^{\text{FR}}+g_{ij}^{\text{FS}}$, where

(58) $$\begin{array}{cc} g_{ij}^{\text{FR}}= & \frac{k_{i}k_{j}\beta^{2}d_{3}^{2}{\text{sech}}^{2}\left(\beta d\right)}{4m^{2}d^{2}},\\ g_{ij}^{\text{FS}}= & \frac{k_{i}k_{j}d_{1}^{2}}{4m^{2}d^{4}}=\frac{k_{i}k_{j}\Delta^{2}}{4m^{2}d^{4}}, \end{array}$$

for i,j=x,y,z. In this model, the QGT contains cross terms and, notably, saturates Inequality Eq. (31):

(59) $$\begin{array}{c} Q_{ii}^{\text{S}}Q_{jj}^{\text{S}}={\left(Q_{ij}^{\text{S}}\right)}^{2}. \end{array}$$

**Fig. 2:**
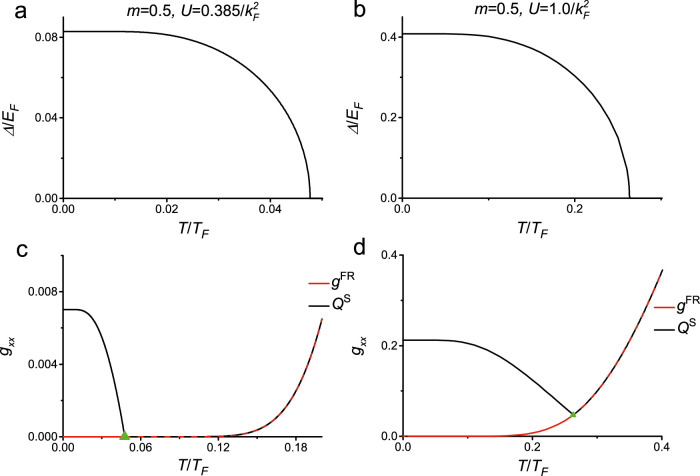
The order parameter Δ as a function of T is shown for $U=0.385/k_{F}^{2}$ (a) and $U=1.0/k_{F}^{2}$ (b). The corresponding temperature-dependent behaviors of the xx-components of $Q^{\text{S}}$ and $g^{\text{FR}}$ are illustrated for $U=0.385/k_{F}^{2}$ (c) and $U=1.0/k_{F}^{2}$ (d), with $k_{x}=k_{y}=k_{z}=1.0k_{F}$.

It is straightforward to verify that the QGT indeed satisfies all the conditions in Eqs. (41) for the equality in Eq. (31) to hold. Unlike the previous models, the imaginary part of the QGT is zero. Additionally, the Fubini-Study contribution to the metric is proportional to $\Delta^{2}$, which vanishes above $T_{c}$. Consequently, the U${}^{N}\left(1\right)$ captures certain signatures of the phase transition in this model. To provide an intuitive understanding of QGT, we present its behaviors as a function of T at $k_{x}=k_{y}=k_{z}=1.0k_{F}$ in Fig. 2, illustrating scenarios with both weak ($U=0.385/k_{F}^{2}$) and relatively strong pairing interactions ($U=1.0/k_{F}^{2}$). Here, m=0.5 and the unit $k_{F}$ represents the Fermi momentum of a non-interacting Fermi gas with the same particle density $n=\frac{k_{F}^{3}}{3\pi^{2}}$, and $T_{F}$ is the associated Fermi temperature. In the top panels of Fig. 2, we plot the order parameter Δ vs T for $U=0.385/k_{F}^{2}$ and $1.0/k_{F}^{2}$ respectively. In the bottom panels, the behaviors of the corresponding QGT and its Fisher-Rao “component”, $g^{\text{FR}}$, are presented. Since all components of QGT are equal at $k_{x}=k_{y}=k_{z}=1.0k_{F}$, as indicated by Eq. (58), we select the xx-components as the representatives to show their features. Above $T_{c}$, Δ=0 and $Q^{\text{S}}=g^{\text{FR}}$. At very low temperatures, $g^{\text{FR}}\to 0$ due to $\lambda_{0}\to 1$ and $\lambda_{n>0}\to 0$. At $T_{c}$, the curve $Q^{\text{S}}\left(T\right)$ is no longer smooth. Interestingly, the geometric quantity QGT encapsulates information about the phase transition, suggesting that the local distance of the quantum mixed state undergoes a non-smooth change as the momentum varies.

## 4 Conclusion

In conclusion, we have established a comprehensive mathematical framework for the U${}^{N}\left(1\right)$ QGT applicable to mixed states, significantly enhancing our understanding of quantum state geometry. By employing the U${}^{N}\left(1\right)$ principal bundle, we introduced the Ehresmann connection, explored the bundle’s fibration, and derived a gauge-invariant metric, along with a Pythagorean-like distance decomposition that highlights the geometric relationships inherent in mixed-state manifolds. Our comparative analysis with the U(1) principal bundle highlights the distinct properties of the U${}^{N}\left(1\right)$ QGT, emphasizing its Riemannian characteristics and the inclusion of a nonzero imaginary part, which is crucial for capturing the complexity of mixed states. Furthermore, the proof of a fundamental inequality for the U${}^{N}\left(1\right)$ QGT reinforces the theoretical foundations. Overall, the work presented here paves a step towards a deeper understanding of the geometric and topological aspects of mixed quantum states.

## Declaration of competing interest

The authors declare that they have no conflicts of interest in this work.

## Appendix A Fundamental inequality for Bures metric

The Bures metric is given by [56]

(A.1) $$\begin{array}{c} g_{\mu \nu }^{\text{B}}=\frac{1}{2}\sum_{ij}\frac{\langle i|\partial_{\mu }\rho |j\rangle \langle j|\partial_{\nu }\rho |i\rangle }{\lambda_{i}+\lambda_{j}}. \end{array}$$

We prove that it also satisfies the inequality

(A.2) $$\begin{array}{c} g_{\mu \mu }^{\text{B}}g_{\nu \nu }^{\text{B}}\geq {\left(g_{\mu \nu }^{\text{B}}\right)}^{2}. \end{array}$$

Proof: Using

$$\begin{array}{c} \partial_{\mu }\rho =\sum_{n}\left[\partial_{\mu }\lambda_{n}\left|n\rangle \langle n\right|+\lambda_{n}(|\partial_{\mu }n\rangle \langle n|+|n\rangle \langle \partial_{\mu }n|)\right], \end{array}$$

we obtain

(A.3) $$\begin{array}{c} \langle m|\partial_{\mu }\rho |n\rangle =\partial_{\mu }\lambda_{n}\delta_{mn}+\left(\lambda_{n}-\lambda_{m}\right)\langle m|\partial_{\mu }n\rangle . \end{array}$$

Substitute it into Eq. (A.1), the Bures metric can be reexpressed as

(A.4) $$g_{\mu \nu }^{\text{B}}=\frac{1}{4}\sum_{n}\frac{\partial_{\mu }\lambda_{n}\partial_{\nu }\lambda_{n}}{\lambda_{n}}+\frac{1}{2}\sum_{nm}\frac{{\left(\lambda_{n}-\lambda_{m}\right)}^{2}}{\lambda_{n}+\lambda_{m}}i\langle m|\partial_{\mu }n\rangle i\langle n|\partial_{\nu }m\rangle ,$$

where in the second item, we have introduced the factor “i” to ensure that $i\langle m|\partial_{\mu }n\rangle$ and $i\langle n|\partial_{\nu }m\rangle$ are both real numbers. For convenience, we let $A_{mn}=\frac{1}{2}\frac{{\left(\lambda_{n}-\lambda_{m}\right)}^{2}}{\lambda_{n}+\lambda_{m}}$, which satisfies $A_{mn}\geq 0$ and $A_{mn}=A_{nm}$. According to Eq. (A.1), we have

(A.5) $$\begin{array}{c} g_{\mu \mu }^{\text{B}}=\sum_{n}{\left(\frac{\partial_{\mu }\lambda_{n}}{2\sqrt{\lambda_{n}}}\right)}^{2}+\sum_{mn}A_{mn}{\left|\langle m|\partial_{\mu }n\rangle \right|}^{2}. \end{array}$$

Thus,

(A.6) $$\begin{array}{cc} g_{\mu \mu }^{\text{B}}g_{\nu \nu }^{\text{B}}= & \sum_{mn}{\left(\frac{\partial_{\mu }\lambda_{n}}{2\sqrt{\lambda_{n}}}\right)}^{2}{\left(\frac{\partial_{\nu }\lambda_{m}}{2\sqrt{\lambda_{m}}}\right)}^{2}\\ + & \sum_{mnkl}A_{mn}A_{kl}|\langle m|\partial_{\mu }n\rangle {|}^{2}{\left|\langle k|\partial_{\nu }l\rangle \right|}^{2}\\ + & \sum_{mnk}[{\left(\frac{\partial_{\mu }\lambda_{k}}{2\sqrt{\lambda_{k}}}\right)}^{2}A_{mn}{\left|\langle m|\partial_{\nu }n\rangle \right|}^{2}\\ + & {\left(\frac{\partial_{\nu }\lambda_{k}}{2\sqrt{\lambda_{k}}}\right)}^{2}A_{mn}{\left|\langle m|\partial_{\mu }n\rangle \right|}^{2}]. \end{array}$$

Applying Cauchy-Schwarz inequality, we obtain

(A.7) $$\begin{array}{cc} & \sum_{mnkl}A_{mn}A_{kl}|\langle m|\partial_{\mu }n\rangle {|}^{2}{\left|\langle k|\partial_{\nu }l\rangle \right|}^{2}\\ & \;\;\geq \sum_{mn}A_{mn}^{2}{\left|\langle m|\partial_{\mu }n\rangle \langle n|\partial_{\nu }m\rangle \right|}^{2}. \end{array}$$

Next, applying Inequality Eq. (34) and the mean value inequality, it can be found

(A.8) $$\begin{array}{cc} & {\left(\frac{\partial_{\mu }\lambda_{k}}{2\sqrt{\lambda_{k}}}\right)}^{2}A_{mn}|\langle m|\partial_{\nu }n\rangle {|}^{2}+{\left(\frac{\partial_{\nu }\lambda_{k}}{2\sqrt{\lambda_{k}}}\right)}^{2}A_{mn}{\left|\langle m|\partial_{\mu }n\rangle \right|}^{2}\\ & \;\;\geq \sum_{k}\frac{\partial_{\mu }\lambda_{k}\partial_{\nu }\lambda_{k}}{2\lambda_{k}}A_{mn}|\langle m|\partial_{\nu }n\rangle ∥\langle m|\partial_{\mu }n\rangle |. \end{array}$$

Combining the above results, we can finally prove Inequality Eq. (A.2).
